# Innate Immune Sensing of DNA

**DOI:** 10.1371/journal.ppat.1001310

**Published:** 2011-04-21

**Authors:** Shruti Sharma, Katherine A. Fitzgerald

**Affiliations:** Division of Infectious Diseases and Immunology, Department of Medicine, University of Massachusetts Medical School, Worcester, Massachusetts, United States of America; University of California San Francisco, United States of America

## DNA Is a Potent Activator of Innate Immunity

When a pathogen attacks, the immune system rapidly mobilizes host defenses in order to reduce the microbial burden and limit damage to the host [1]. Innate immunity is the first line of defense and relies on germ line–encoded pattern recognition receptors (PRRs) such as the Toll-like receptors (TLRs), which sense microbial products that are not normally found on or in mammalian cells. The considerable potency of nucleic acids as triggers of the innate immune response has gained appreciation over the last few years. In particular, nucleic acid sensing of viruses is central to anti-viral defenses through recognition of viral genomes or nucleic acids generated during viral replication. Distinct classes of nucleic acid sensing molecules have been uncovered that function in different cell types and subcellular compartments to coordinate innate defenses (reviewed in [2]).

While recognition of RNA molecules is dependent on members of the TLR family and cytosolic RNA helicases, the mechanisms underlying the sensing of DNA have been less well defined. It has been known for over a decade that DNA, the most recognizable unit of life, is a potent trigger of inflammatory responses in cells. The discovery of TLR-9, a receptor for hypomethylated CpG-rich DNA, partially explained these findings [3]. TLR9 is localized to the endosomal compartment and in humans is expressed in B cells as well as in plasmacytoid dendritic cells (pDCs). However, it became clear that the immune stimulatory activity of microbial DNA was not compromised in many cells lacking TLR9 [4]. These observations prompted new efforts to understand how DNA triggers immune responses, an endeavor that has led to the discovery of several new DNA recognition receptors and fresh insights into infectious as well as autoimmune diseases.

## There Are Multiple Receptors for Microbial DNA

A significant effort from many laboratories has highlighted the importance of cytosolic DNA sensing in the innate immune response. At least six intracellular receptors have been implicated to some degree. These include DNA-dependent activator of interferon (IFN)-regulatory factors (DAI) (also called Z-DNA-binding protein 1, ZBP1) [5], absent in melanoma 2 (AIM2) [6]–[9], RNA polymerase III (Pol III) [10], [11], leucine-rich repeat (in Flightless I) interacting protein-1 (Lrrfip1) [12], DExD/H box helicases (DHX9 and DHX36) [13], and most recently, the IFN-inducible protein IFI16 [14]. DAI was the first to be implicated in synthetic B- and Z-form dsDNA recognition [5]; however, the role of DAI is still unclear, as DAI-deficient mice and cells coordinate normal immune responses to DNA [15]. Cytoplasmic dsDNA also triggers IFN production via RNA Pol III, which transcribes the DNA into 5′-ppp RNA, a ligand for the RNA helicase RIG-I [10], [11]. In pDCs, DHX9 and DHX36 contribute to cytosolic CpG-DNA and HSV-1-driven IFN responses [13], which likely account for previously reported TLR9-independent cytokine responses to some DNA viruses [12]. Lrrfip1 appears to bind both DNA and RNA; however, Lrrfip1 does not regulate the transcription factors that drive IFN gene transcription, but rather signals a co-activator pathway involving β-catenin and CBP/p300 histone modifying complexes to enhance the transcription of type I IFNs in the nucleus [16]. DNA from *Listeria monocytogenes* and RNA from vesicular stomatitis viral (VSV) activate this Lrrfip1-β-catenin pathway to mediate these effects.

Immune responses to DNA are not restricted to type I IFN-inducing pathways: cytosolic DNA also activates caspase-1-dependent maturation of the pro-inflammatory cytokines interleukin (IL)-1β and IL-18. This pathway is mediated by AIM2, a PYHIN (Pyrin- and HIN200-domain-containing) protein. Recent evidence from knockout studies has revealed the importance of AIM2 in host defense to cytosolic bacteria such as *Fransicella* spp., as well as DNA viruses like mouse cytomegalovirus (reviewed in [17]–[20]). The newest receptor identified, IFI16, binds viral DNA and is critical in the immune response to certain DNA viruses [14]. Like AIM2, IFI16 is a PYHIN protein that binds viral DNA via HIN domains; however, IFI16 does not appear to associate with ASC to regulate IL-1β maturation. Rather, IFI16 activation induces IFN-β and inflammatory cytokine production in response to cytosolically administered viral DNA or HSV1 infection.

## Distinct Classes of DNA Sensors Engage Distinct Signaling Complexes

Most of these DNA sensors utilize a subset of adapter molecules, which relay signals to NF-κB and members of the interferon regulatory factor (IRF) family. TLR9 as well as DHX9 and DHX36 recruit MyD88 to activate IFN production in pDCs in response to DNA. In contrast, recognition of DNA by RNA-Pol III generates an RNA intermediate, which signals via RIG-I and MAVS. In the case of IFI16, the endoplasmic reticulum–resident protein stimulator of interferon genes (STING) relays signaling downstream [21]. Whether STING binds IFI16 directly or merely acts as a signaling intermediate for this pathway is unclear. AIM2 triggers caspase-1 activation via the PYD domain containing adapter molecule ASC. Although IFI16 also contains a PYD domain, it does not appear to utilize ASC for IFN production. It is likely that the DAI pathway also involves STING, although this has not been formally demonstrated. Downstream of STING, MAVS, or MyD88, the nucleic acid sensing pathways converge on different IKK kinases to phosphorylate and activate IRFs (reviewed in [2]). In the case of the TLRs and possibly DHX helicases, IKKα is involved in phosphorylating IRF7, while downstream of MAVS and STING, TANK-binding kinase 1 (TBK-1), an IKK-related kinase, phosphorylates and activates IRF3. There is no evidence for the involvement of adaptor proteins in the Lrrfip1-β-catenin pathway, although intermediary-signaling molecules may be required for Lrrfip1-dependent β-catenin phosphorylation.

## Cytosolic DNA Recognition Pathways Also Contribute to the Pathogenesis of Autoimmune Disease

While DNA recognition receptors and associated signaling pathways are part of the normal immune response to infection, self DNA that gains access to compartments where these sensors are localized can also trigger inflammation, with deleterious consequences for the host (reviewed in [1]). Systemic lupus erythematosis (SLE) is one of the first autoimmune diseases where aberrant self-DNA recognition and type I IFNs play a role in disease pathogenesis. DNA and RNA complexed with autoantibodies trigger immune activation, leading to autoantibody production and significant cell death. Here, TLR7- and TLR9-sensing pathways in autoreactive B cells and pDCs appear to be central to disease pathogenesis [1]. Mutations in enzymes that normally degrade DNA have been linked to SLE and other diseases. For example, defective clearance of extracellular nucleic acids from dying cells due to deficiency or mutation of DNAse I causes a lupus-like syndrome in mice and humans [22], [23]. The sensing of accumulated DNAse I substrates is unclear but likely involves TLRs as well as other DNA sensors.

DNases regulate the accumulation of DNA in more than one compartment. For instance, DNase II is localized to lysosomes where it normally degrades DNA from engulfed apoptotic and necrotic cells. Interestingly, DNAse II–deficient mice are embryonic lethal due to overproduction of type I IFNs [24], [25]. However, mice deficient in both DNAse II and the type I IFN receptor are viable. The DNA sensing mechanism triggering IFN in this case is known to be TLR independent but dependent on IRF3 and IRF7. It is likely that one or more of the DNA sensors described above account for these responses. Another type of deoxyribonuclease, DNAse III, also called 3′ repair exonuclease 1 (TREX1), is found on the endoplasmic reticulum and has been shown to digest cell-intrinisic DNA generated as a result of reverse transcription from endogenous retroelements. Under normal circumstances TREX1 prevents the accumulation of this reverse transcribed DNA [26]. However, in situations where TREX1 is non-functional, DNA accumulates and can lead to activation of cytosolic sensing pathways. Mutations in TREX1 are found in patients with Aicardi-Goutières syndrome (AGS) and chilblain lupus, diseases that clinically resemble congenital viral infections [26], [27]. Mutations in the sterile a motif (SAM domain) and HD domain-containing protein 1 (SAMHD1) are also linked to this disease [27], [28]. Although there is no direct evidence linking SAMHD1 to cytosolic DNA sensing per se, it is likely that SAMHD1 also acts to counterbalance cytosolic DNA sensing and/or signaling, perhaps by interfering with one or more of the sensors above.

## There Are Still Major Unknowns in the World of DNA Sensing

Fresh new insights into infectious as well as autoimmune diseases have been gained as a result of the studies on DNA sensing and signaling pathways. While there has been great progress in this area, many important questions arise from these discoveries. How these different sensors coordinate cell type–specific and or species-specific responses to DNA is still a major question and undoubtedly the focus of future research efforts in this area. Another key issue to be resolved is how DNA ligands, which are often enclosed in membrane-bound compartments (e.g., DNA viruses replicating in the nucleus), meet these cytosolic receptors. The identification of TREX1 as well as SAMHD1 suggests that in healthy cells, tightly controlled DNA levels prevent engagement of these pathways. It is likely that additional counter regulatory mechanisms that dampen these responses will be uncovered. Moreover, it is also likely that future discoveries will unveil mechanisms by which pathogens inactivate these defenses to prevent the immune response from sampling their genomes and turning on anti-viral defenses. Further characterization of these DNA sensing and counter regulatory mechanisms is likely to impact our understanding of common autoimmune and autoinflammatory diseases as well as build a framework for our understanding of infectious diseases. Future discoveries in this area will no doubt unveil new opportunities for therapeutic interventions in infectious and autoimmune disease.[Fig ppat-1001310-g001]

**Figure 1 ppat-1001310-g001:**
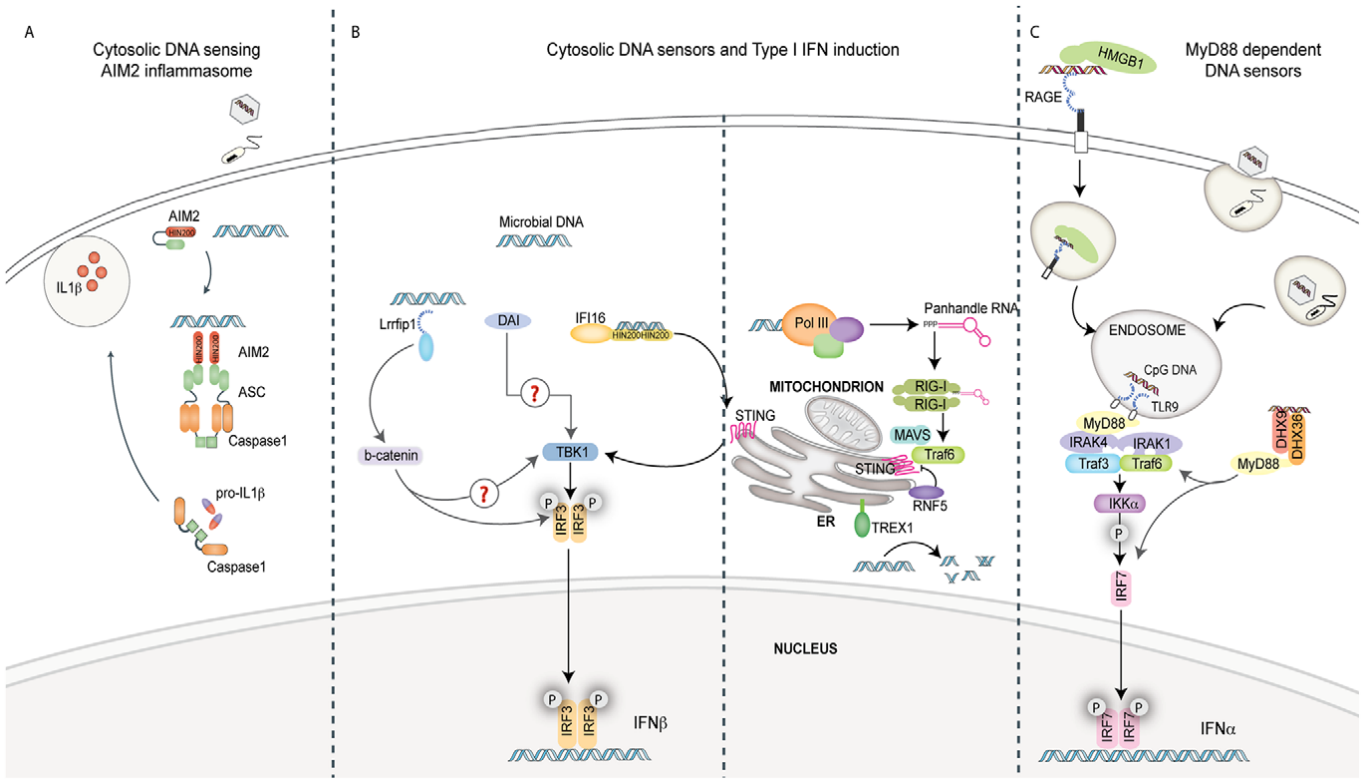
Pathways of innate immune sensing of DNA. (A) Cytosolic DNA from invading viruses and bacteria engage and activate AIM2 binding to the adaptor ASC. ASC mediates caspase-1-dependent pro-IL-1β/pro-IL-18 cleavage and secretion of their bioactive forms, IL-1β and IL-18. IL-1β and IL-18 are significant mediators of inflammatory responses to infection. (B) Four known cytosolic sensors are represented here. Lrrfip1 recognized viral DNA as well as RNA to induce IFNβ via a β-catenin-IRF3 transactivator pathway independently of the kinase TBK1. DAI can bind double-stranded B-form and atypical Z-form DNA to induce TBK1-IRF3-dependent IFNβ production. Evidence for the role of adaptors MAVS/STING in these pathways is lacking. IFI16 can directly bind viral DNA via its HIN200 domains and initiate IFNβ induction in a STING-TBK1- and IRF3-dependent manner. RNA polymerase III (Pol III) generates 5′ tri-phosphate RNA that is a ligand for RIG-I. RIG-I signals via the adaptor MAVS, subsequently activating ubiquitin ligase TRAF3 and subsequently TBK1 and IRF3. The ubiquitin binding protein RNF5 inhibits STING activation by targeting it to the proteasome, while TREX1 inhibits/prevents IFNβ production by degrading DNA substrate. (C) The receptor for advanced glycated end products (RAGE) and HMGB1 can bind extracellular CpG-rich DNA and transport it to a TLR9-positive compartment. Here, it is recognized by TLR9 and signals via MyD88 and the IKK kinase, IKKα, and IRF7 in pDCs to induce IFNα production. The cytosolic DExD/H box helicases DHX9/DHX36 can recognize cytosolic CpG DNA and initiate signaling to IRF7 via MyD88.
